# SARS-CoV-2-associated lymphopenia: possible mechanisms and the role of CD147

**DOI:** 10.1186/s12964-024-01718-3

**Published:** 2024-07-04

**Authors:** Shaimaa Shouman, Nada El-Kholy, Alaa E. Hussien, Azza M. El-Derby, Shireen Magdy, Ahmed M. Abou-Shanab, Ahmed O. Elmehrath, Ahmad Abdelwaly, Mohamed Helal, Nagwa El-Badri

**Affiliations:** 1 Center of Excellence for Stem Cells and Regenerative Medicine, Zewail City of Science and Technology, Giza, 12587 Egypt; 2 Biomedical Sciences Program, University of Science and Technology, Zewail City of Science and Technology, Giza, 12587 Egypt; 3 Medicinal Chemistry Department, Faculty of Pharmacy, Suez Canal University, Ismailia, 41522 Egypt; 4 Institute for Computational Molecular Science, Department of Chemistry, Temple University, Philadelphia, PA 19122 USA; 5 Department of Drug Discovery, H. Lee Moffit Cancer Center& Research Institute, Tampa, FL 33612 USA; 6 Cancer Chemical Biology Ph.D. Program, University of South Florida, Tampa, FL 33620 USA; 7 Faculty of Medicine, Cairo University, Cairo, Egypt

**Keywords:** Lymphopenia, T-cells SARS-CoV-2, COVID-19, CD147 receptor

## Abstract

T lymphocytes play a primary role in the adaptive antiviral immunity. Both lymphocytosis and lymphopenia were found to be associated with severe acute respiratory syndrome coronavirus 2 (SARS-CoV-2). While lymphocytosis indicates an active anti-viral response, lymphopenia is a sign of poor prognosis. T-cells, in essence, rarely express ACE2 receptors, making the cause of cell depletion enigmatic. Moreover, emerging strains posed an immunological challenge, potentially alarming for the next pandemic. Herein, we review how possible indirect and direct key mechanisms could contribute to SARS-CoV-2-associated-lymphopenia. The fundamental mechanism is the inflammatory cytokine storm elicited by viral infection, which alters the host cell metabolism into a more acidic state. This “hyperlactic acidemia” together with the cytokine storm suppresses T-cell proliferation and triggers intrinsic/extrinsic apoptosis. SARS-CoV-2 infection also results in a shift from steady-state hematopoiesis to stress hematopoiesis. Even with low ACE2 expression, the presence of cholesterol-rich lipid rafts on activated T-cells may enhance viral entry and syncytia formation. Finally, direct viral infection of lymphocytes may indicate the participation of other receptors or auxiliary proteins on T-cells, that can work alone or in concert with other mechanisms. Therefore, we address the role of CD147―a novel route―for SARS-CoV-2 and its new variants. CD147 is not only expressed on T-cells, but it also interacts with other co-partners to orchestrate various biological processes. Given these features, CD147 is an appealing candidate for viral pathogenicity. Understanding the molecular and cellular mechanisms behind SARS-CoV-2-associated-lymphopenia will aid in the discovery of potential therapeutic targets to improve the resilience of our immune system against this rapidly evolving virus.

**Graphical Abstract:**

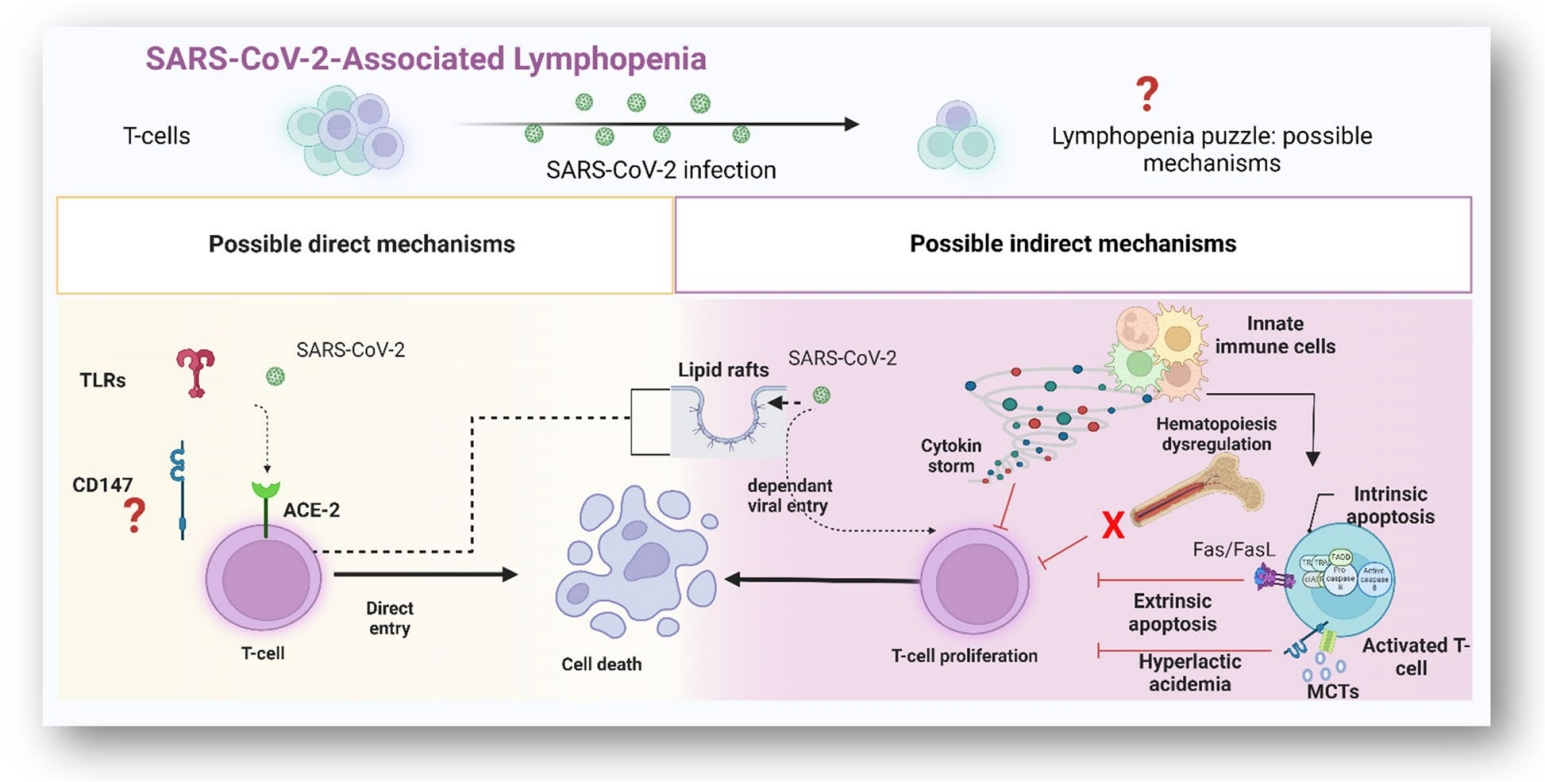

## Introduction

**Fig. 1:**
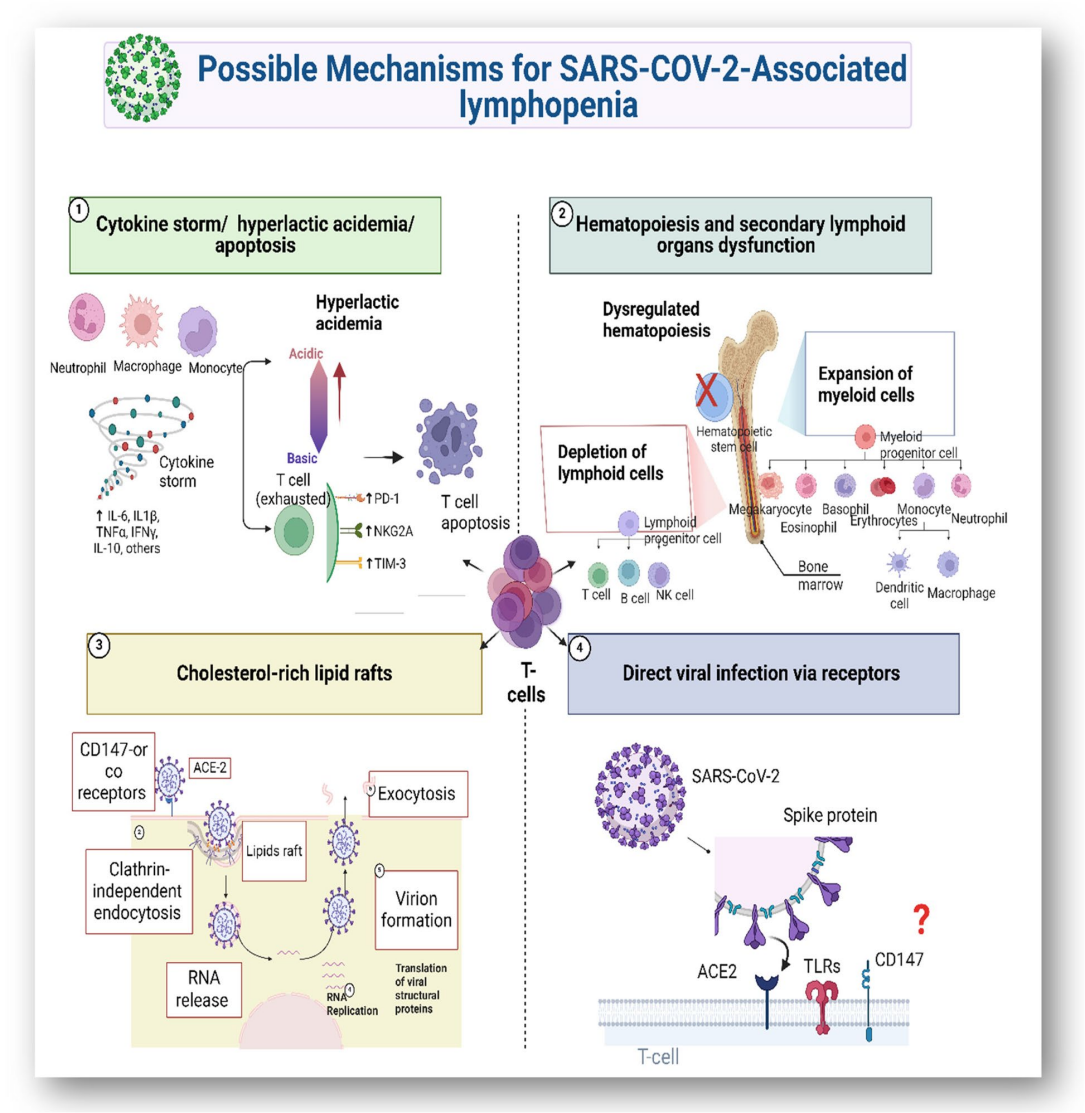
Schematic representation of possible mechanisms of lymphopenia in SARS-CoV-2 viral infection

In 2019, the outbreak of novel severe acute respiratory syndrome coronavirus-2 (SARS-CoV-2) rapidly became a global predicament. SARS-CoV-2, like other RNA viruses, is vulnerable to mutations over time, leading to the emergence of new variants. Some of these variants exhibited changes in transmissibility, severity, and in some cases, resistance to immunity caused by prior infection or vaccination [1, 2]. Although SARS-CoV-2 infection triggers host immunity, the primary immunopathological feature are uncontrolled inflammatory innate and inadequate adaptive immune responses. Patients with severe -but not with low or mild condition showed a low lymphocytes count. In particular, the numbers of CD4^+^ T-cells, CD8^+^ T-cells, CD3^+^ T-cells, CD19^+^ B-cells, and natural killer (NK) were substantially reduced [3]. Furthermore, aberrant interferon (IFN) production, cytokine storms, and ineffective or delayed neutralizing antibody induction are co-currently observed [4]. The virus invades cells primarily through the interaction of its spike protein with angiotensin-converting enzyme 2 (ACE2) receptors. However, some immune cells, particularly CD4^+^ and CD8^+^ T-cells, scarcely express ACE2 [5, 6], prompting many questions concerning the cause. In this review, we summarize the possible underlying mechanisms of SARS-CoV-2-associated-lymphopenia (Fig. 1). In response to viral infection, immune cells secrete various pro-inflammatory cytokines including IL-1, IL-6, interferon (IFN)-γ, and tumor necrosis factor (TNF), shifting the metabolic state toward aerobic glycolysis [7–9]. High concentrations of lactate byproducts, resulting in lactic acidosis, which in favor of virus replication but, probably, not for lymphocytes proliferation [10, 11]. Cytokine storm and metabolic shift acts as an indirect mechanisms for promoting T lymphocyte depletion and, most likely, death by activating certain intrinsic and/or extrinsic apoptotic pathways [12]. Another proposed mechanism is that, a cholesterol-rich lipid raft on an activated T-cells membrane offer a platform to facilitate viral entrance [13, 14]. Indirect damage of these lipids microdomains specifically inhibits T lymphocyte proliferation and might contribute in this depressed cell count [15]. Moreover, the main site of hematopoiesis in the bone marrow (BM) is directly or indirectly perturbed by SARS-CoV-2 infection. The BM of highly infected patients has lymphoid progenitor depletion, while immature granulocyte-monocyte progenitor cells (GMPs) accumulate [16, 17]. Secondary lymphoid organs, on the other hand, suffer substantial tissue damage such as lymph follicle depletion, splenic nodule shrinkage, histiocyte hyperplasia, and lymphocyte decreases as a result of SARS-CoV-2 infecting mainly tissue-resident CD169^+^ macrophages [18]. In addition to these indirect mechanisms, direct killing of T lymphocytes is also a possible event. This is achieved either via ACE2, CD147, CD26, LFA-1, neuropilin 1 (NRP1) and/or toll-like (TLRs) receptors, expressed mainly on activated T-cells [19–22].These abovementioned mechanisms are not mutually exclusive and can work together resulting in lymphopenia progression in SARS-CoV-2 infection. The role of CD147 receptors is also worth exploring as they are not only expressed on T lymphocytes [23]. They were also reported as novel receptors involved in the entry of SARS-CoV-2, as well as several pathogens and emerging mutant viral strains into the host cells [24, 25]. This CD147 could be descripted as pleiotropic molecule due to its ability to interact and regulate cyclophillin (Cyps) [26], matrix metalloproteinases (MMPs) [27], and monocarboxylate transporters (MCTs) [28], to mediate various cellular processes [29]. Each co-partner participates in a distinct route that would facilitate virus infection. MPPs stimulate cell-to-cell fusion, which enhance virus dissemination. The CypA/CD147 interaction mediates intracellular downstream pathways involved in inflammation. CD147 is necessary for MCTs translocation, to allow energy production relies more heavily on glycolysis. Altogether, this makes CD147 plays a crucial role in the pathogenesis of SARS-CoV-2 infection.

## Lymphopenia

### Etiology, diagnostic, and prognostic significance

Since 1970, Bone and Lauder have identified lymphopenia as an undesirable predictor in individuals with advanced cancer [30]. Lymphocytes count can be as low as (1 × 10 ^9^/L) in adult and (3 × 10 ^9^/L) in children [31, 32]. Both T-cell and B-cell numbers decline however, natural killer (NK) cell lymphocytopenia is uncommon. The neutrophil-to-lymphocyte ratio (NLR) can also be utilized as a measure of systemic inflammation. NLR is a good predictive marker of immune response to various infectious and non-infectious diseases [33]. Modulation of other T-cells subpopulations has been described including increased frequency of Tregs, Th17 cells, and PD-L1 ^+^ T-cells [34, 35]. The majority of these changes were associated with a poor prognosis, but they are not directly related to lymphopenia [36]. The etiology of lymphopenia in cancer was thought to be caused by T lymphocytes redistribution and interaction of myeloid-derived suppressor cells (MDSC) with T-cells and block their proliferation [37]. In general, the etiology of low lymphocyte count could be attributed to insufficient production of the lymphocytes from the bone marrow, or their destruction due to infection, autoimmune diseases, radiation therapy, metabolic disorders, inflammation, stress, obesity, diabetes, aging, malnutrition and congenital conditions [38–41]. In infectious diseases, lymphocyte destruction and diminished production were also reported in *Mycobacterium tuberculosis* (M. tuberculosis) and *plasmodium falciparum malari*a. Similar mechanism was postulated to be correlated with the reallocation of T lymphocytes in the tissues [42]. Besides that, M. tuberculosis-induced T lymphocyte apoptosis, hematopoietic dysfunction, and T-cell exhaustion [43]. While malaria-associated lymphopenia was described to be mainly due to Fas-induced apoptosis [44], other studies reported no detected apoptosis in the peripheral blood mononuclear cells (PBMCs) [45]. In viruses, most of them lead to relative lymphocytosis, while only a few ones cause lymphopenia. For instance, lymphopenia and/or monocytosis were reported as markers for the influenza A virus subtype H1N1 [46]. Other viruses including rubeola, human immunodeficiency virus (HIV), polioviruses, and varicella-zoster can directly infect lymphocytes leading to their destruction and shortage in their production [47, 48]. The exposure of lymphocytes from different subpopulations to influenza A virus was found to induce apoptosis in CD3^+^, CD4^+^, CD8^+^, and CD19^+^ lymphocytes through the Fas-FasL signaling pathway [49]. Pro-apoptotic molecules such as FasL, TNF-α, and TRAIL were up-regulated in chronic hepatitis C virus (HCV) infection, propounding the immune cell death via the intrinsic and extrinsic pathways [50, 51]. HIV is another infectious disease that is associated with CD4^+^ lymphopenia leading to significant immunodeficiency, where CD4^+^ lymphocytes are either directly infected or become unfunctional [52]. CD4^+^ T-cells from HIV patients exhibited modifications in the microdomains of their cell membranes that impaired their functionality. T-cell unresponsiveness was attributed to high plasma levels of phospholipase A2 group IB (PLA2G1B), either co-dependent with HIV gp41 protein or independently at high concentrations. PLA2G1B also decreased the survival and proliferation rate of the CD4^+^T-cell [53]. Whether there are similar alterations combined with SARS-CoV-2 infection, remains unknown. T-cell depletion in peripheral blood is reported in up to 85% of severe COVID-19 patients [54]. Studies on severe COVID-19 cases reported exhaustion of T-cells, high expression levels of programmed cell death protein 1 (PD-1), T-cell immunoglobulin and mucin domain 3 (Tim-3), and NKG2A along with the diminished expression of T-cell activation markers CD107, and interferon gamma **(**IFN-γ) [41]. A significant decrease in lymphocyte count was observed either in CD8^+^ cell alone [55] or in both CD4^+^ and CD8^+^cells [56], especially in intensive care unit (ICU) COVID-19 patients. However, a decrease in the B lymphocyte count was also observed in COVID-19 patients, albeit inconsistently [55, 57]. Managing the underlying cause is typically necessary while treating lymphopenia. Although lymphopenia is a symptom rather than a disease, understanding the mechanism underlying this immunological change is critical to the effectiveness of vaccination strategies. Possible mechanisms are described further below.

## Possible mechanisms for lymphopenia in SARS-CoV-2 patients

### Indirect mechanisms

#### Inflammatory cytokines storms and lymphopenia

The heavily SARS-CoV-2 viral load in severely infected patients causes over-stimulation and excessive infiltration of immune cells like macrophages, neutrophils, and reactive T-cells to shed out the virus. This process however may fail to eliminate the virus, and instead, causes immune cell exhaustion and over-production of pro-inflammatory cytokines, resulting in multi-organ failure, and leading to acute respiratory distress syndrome (ARDS) [58, 59]. Both lymphopenia and high pro-inflammatory cytokines (cytokine storm syndrome, CSS) were reported to be associated with moderate to severe ICU hospitalized cases [60, 61]. Interleukin 6 (IL-6) is a pro-inflammatory cytokine produced mainly by activated macrophages during acute inflammation. It promotes the activation and proliferation of CD4^ +^ cells during chronic inflammation [62–64]. Although IL-6 activates other immune cells during the inflammatory response, it is overproduction may lead to the abortion of lymphopoiesis. This mechanism was proposed to be due to the suppression of lymphoid lineage commitment while promoting the expansion and survival of the undifferentiated myeloid progenitors [65, 66]. Despite that, innate immune cells produce multiple inflammatory cytokines (IL-6, IL-18 and IL-33,IL-1, IL-1β IL-10, IL-8, IL-17, CXCL10, CCL2, CXCL9, TNF-α, and IFN-γ) [54, 67], the only combination of IFN-γ and TNF-α promote inflammatory cell death [68]. Co-expression of IFN-γ and TNF-α activates Janus kinase/signal transducer and activator of transcription (JAK/STAT) signaling pathway that induces downstream nitric oxide (NO) production and firing down caspase-8/FADD-mediated PANoptosis [68, 69].TNF-α is the master regulator of pro-inflammatory cytokines production [70] and plays a central role in cell necrosis and apoptosis [71, 72]. Although the exact mechanism of TNF-α associated lymphopenia is still unknown, the cytokine was reported to promote lympholysis, increase the adhesion of the immune cells to the endothelial surface, and enhance the recruitment of lymphocytes to the lymphoid and non-lymphoid organs [73–75].Lymphopenia was reported to be combined with neutrophilia, especially in patients suffering from CSS [76]. IFN-γ may play an important role in this neutrophil proliferation, as several studies showed its increased production from activated neutrophils during bacterial infection [77–79]Interestingly, IFN-γ, but not IFN-α or IFN-β stimulated neutrophils to acquire the ability to inhibit lymphocyte proliferation through the expression of programmed death ligand 1 (PD-L1) [80], which may explain the high count of neutrophils associated with overproduction of IFN-γ and lymphopenia [81–83]. Another suggested reason for T-cell exhaustion was mediated by macrophages via cytokine-receptor axes such as CCL2-CCR2, CCL3-CCR1, CCL3-CCR5, CCL4L2-VSIR, and CCL4-CCR5 [84].However, these rapidly dividing ‘first-line’ immune cells like macrophages and dendritic cells (DCs) can indeed influence the cellular metabolism to meet their high energetic and biosynthetic demand. Many studies suggest that inflammatory conditions shift the cellular metabolism towards a more glycolytic state [85, 86]. This increased reliance on glycolysis can lead to the accumulation of lactate, resulting in more acidic condition, as described below. Upon viral infection, macrophages are activated by TLRs or primed by IFNγ. Macrophages undergo aerobic glycolysis to facilitate elevated secretion of inflammatory mediators [87, 88]. This metabolic shift also triggers a cascade of signaling events, leading to various cellular responses including PI3K/AKT–dependent DCs maturation [89], mTORC1-dependent NKs stimulation [90], phorbol 12-myristate 13-acetate-activated neutrophils [91, 92], B cells [93], and effector T-cells including both CD4^+^ and CD8^+^ T-cells activation. Early glycolytic phase was significant for rapid IFN-γ production by memory CD8^+^ T-cells to recruit more macrophages resulting in a vicious CSS cycle [94].Even though the glucose transport facilitators expression increased, such as glucose transporter type 1 (GLUT1), glucose availability is insufficient [95]. Effector cells can quickly adjust to low glucose levels by boosting their uptake of glutamine and starting glutaminolysis to maintain the TCA cycle [96]. Furthermore, when glutamine availability is disrupted, effector T-cells development and function are disturbed, causing these cells to transition from CD4^+^T helper cells to a Treg cells phenotype [97]. However, aggressive inflammatory CSS cannot be calmed by Treg cells via the production of IL-10 and tumor growth factor-β (TGF-β) [98]. As results, metabolic reprograming of immune cells towards aerobic glycolysis allows these cells to cope better with metabolically restricted inflammatory microenvironment, especially those seen under hypoxic conditions [99]. However, SARS-CoV-2, on the other hand, takes advantage of this condition, and its ORF3a protein increased hypoxia-inducible factor (HIF-1) expression by triggering mitochondrial-derived reactive oxygen species (ROS) damage. HIF-1α subsequently enhanced viral infection and pro-inflammatory cytokines production, especially in elderly patients [100], who usually suffer more severe inflammatory responses and may be at higher risk of COVID-19-associated death [101]. However, the ability of stimulated T-cells under these hypoxic conditions to proliferate was reduced, despite increased expression of their CD25 (IL-2 receptor) [99]. This could be explained that pathophysiological hypoxia affects T-cell proliferation and viability via disturbed IL-2R signalling downstream of STAT5a phosphorylation, but not as a result of impaired cellular energy homeostasis. Collectively, impaired fine-tuning of the immune response was advantageous to the virus survival but not to lymphocyte.

#### Inhibition of lymphocytes by hyperlactic acidemia

**Fig. 2:**
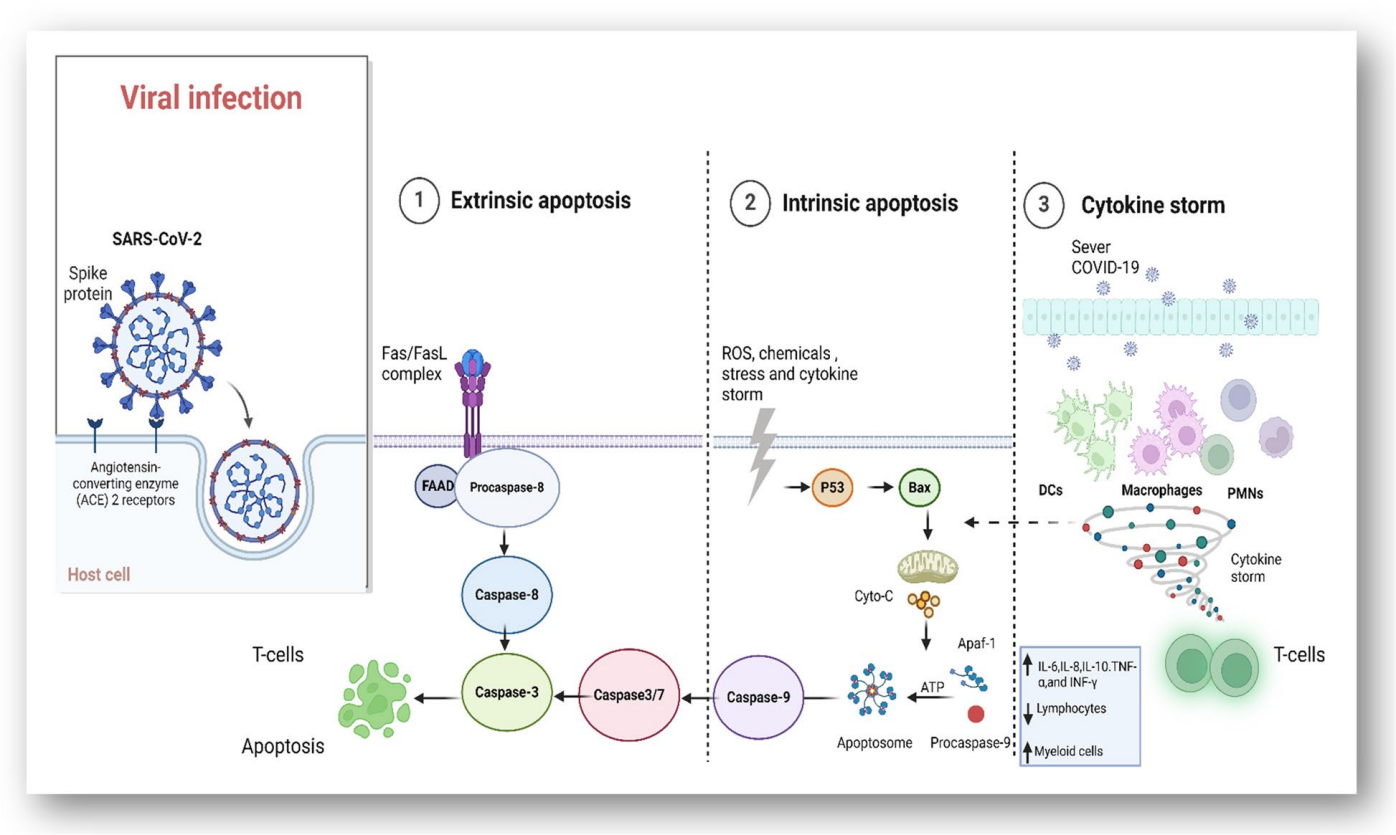
Indirect mechanisms of SARS-CoV-2 associated lymphopenia: (1) FASL expressed on cytotoxic T-lymphocytes (CTLs) activation of the extrinsic pathway of FAS. Fas-associated death domain (FADD) adaptor with pro–caspase-8 modulates caspase-8 activation. The activation of caspase-8 regulates the proteolytic cleavage of caspase-3 and − 7, priming the extrinsic pathway of apoptosis. (2) The intrinsic apoptotic pathway is triggered by various cellular stress pathways such as oxidative stress, cytotoxic chemicals, hypoxia, ER stress, or/and DNA damage. (3) COVID-19 patients cytokine storm is derived by recognition of viral nucleic acids through TLRs in the endosome [139]

It has become increasingly clear that viruses could alter the host cell metabolism. Viruses have evolved a variety of strategies to serve their needs and also limit host immune response to ensure their survival [102, 103]. Numerous non-mitochondrial processes that support macromolecular synthesis, such as those contribute to the production of viral nucleotides and proteins, could be utilized by virus [104]. The end product of glycolysis is lactate, even in the presence of oxygen. Elevated plasma lactate level in severely infected COVID-19 patients was reported to cause metabolic acidosis with multiple-organ failure [10, 105, 106]. Metabolomics study at early time point around 8 h after SARS-CoV-2 infection, showed marked lower intracellular glucose levels and high lactate levels, which confirm the priming of glycolysis to support the strong demand for viral replication [107]. The infection causes the generation of mitochondrial ROS, which leads to the stabilization of the HIF-1 and, as a result, stimulation of glycolysis. HIF-1α-induced glycolysis, and the consequent pro-inflammatory monocytes stimulation inhibit T-cell response [10, 108]. Although low ROS concentrations are a prerequisite for T-cell survival, upregulated ROS release from activated monocyte macrophages may drive T-cell apoptosis [109, 110]. A positive feedback loop is initiated from this uncontrolled oxidative stress that triggers NLRP3-inflammasome activation, pyroptosis, and the production of more pro-inflammatory mediators [111, 112]. Acidosis and hypoxia mutually reinforce one another and were reported to be linked to poor clinical outcomes in COVID patients, and blockade of glycolysis by 2-DG was reported to prevent SARS-CoV-2 replication [113]. Although lymphocyte proliferation may be inhibited and lead to death by elevated blood lactic acid levels [11, 114], the molecular mechanisms underlying this response are not resolved yet. A novel route for SARS-CoV-2 invasion, CD147 was reported to have a central role in controlling the export of lactic acid molecules outside the cell (Fig. 2). CD147 interacts with MCTs, which act as co-transporters of protons and lactate anions down a concentration gradient [115]. MCT stability and localization within the plasma cell membrane are controlled by ancillary chaperone glycoproteins through physical interaction. Lack of those chaperones leads to the accumulation of MCTs within the cell [28, 116, 117]. CD147 acts as the major chaperone protein for MCT 1, MCT 3, and MCT 4 [118, 119]. For example, CD147 was found to play a major role in tumorigenesis by increasing lactate export through MCT localization [120]. This allows tumor cells to cope with the increased glycolysis and lactate levels [121, 122]. Similar to cancer cells, activated effector T-cells have been reported to undergo increased glycolysis and lactate production, while memory T-cells utilize the oxidative phosphorylation (OXPHOS) machinery [123, 124], as they need high energy supplies for proliferation and cytokine production [125]. T-cells also express MCTs for lactate export [126]. Despite the reported interaction of CD147 with MCTs more data are required to determine the effect of CD147-binding on MCT stability and localization, and its role in controlling the hyperlactic acidemia prevalent in SARS-CoV-2 infected patients.

#### Activation of apoptotic pathway of virus-infected T-lymphocyte

Activation of apoptotic pathways is one of the indirect mechanisms for T-cell death in SARS-CoV-2 pathogenicity. However, so far, the question of whether SARS-CoV-2 can directly cause T-cells apoptosis remains to be explored. Consistent findings in other enveloped viruses, such as murine hepatitis virus (MHV), HIV, and avian infectious bronchitis virus have also shown T-cells depletion via apoptosis [127]. Concordant with the emergence of severe acute respiratory syndrome, lymphopenia was proposed to occur due to up-regulation of genes responsible for apoptosis as demonstrated in human PBMCs infected with SARS-CoV [127]. Programed cell death is initiated by activating either intrinsic or extrinsic pathways which lead to a series of downstream cascades of events (Fig. 2). The extrinsic pathway involves activation of cell-surface death receptors like Fas/CD95 by its ligands, while the intrinsic pathway involves activation of BCL-2-family proteins leading to permeabilization of the mitochondrial outer membrane and cell death. Both the intrinsic and extrinsic pathways lead to the activation of a family of proteases, such as caspases [128, 129].Over-expression of 3a non-structural viral protein (NSP3) in SARS-CoV induced a caspase-8 -dependent apoptosis in Vero E6 cells [127]. SARS-CoV E protein was shown to be responsible for the induction of apoptosis in transfected Jurkat T-cells. A reported interaction between SARS-CoV E protein (BH3-like region in the C-terminal cytosolic domain) and BclxL antiapoptotic protein (BH3 domain) proposed a sequestration model. This interaction prevented the inhibition of the pro-apoptotic pathway by BclxL suggesting its importance in SARS-CoV-induced lymphopenia [130]. Overexpression of BclxL inhibited apoptosis by blocking the activation and translocation of the pro-apoptotic proteins Bax and BH3 from the cytosol to mitochondria and inhibiting the subsequent cytochrome c release and activation of caspases [130]. A study by Boonnak et al. showed that influenza-specific CD8^+^ T-cells elicited Fas/FasL mediated T-cell cytotoxic response in the highly pathogenic H5N1 viral infection. However, the activated T-cells were also susceptible to the same antiviral destruction mechanism as they also expressed the Fas antigen. The highly virulent H5N1 virus takes advantage of this mechanism by promoting the expression of FasL on plasmacytoid dendritic cells leading to T-cell apoptosis and subsequent lymphopenia [131]. Fas antigen is type-II transmembrane protein that belongs to the TNF receptor family [132, 133], and binding with cognate Fas ligand mediates the transduction of apoptotic signals in activated T-cells [134]. Influenza A virus was also reported to induce apoptosis in peripheral blood lymphocytes by the same Fas-FasL apoptotic pathway [49]. MERS-CoV virus infects T- cells through the highly expressed receptor dipeptidyl peptidase 4 (DPP4). Both the intrinsic and extrinsic pathways were implicated in T-cell apoptosis upon MERS-CoV infection, especially in the spleen and tonsils [135]. In a recent study, forty-two Caucasian SARS-CoV-2 patients were reported to express higher levels of Fas and PD-1, possibly resulting in T-cell death and exhaustion, and accounting for lymphopenia [12]. Moreover, extensive T-cell apoptosis was found in COVID-19 patients with poor prognosis and was positively correlated with increased plasma levels of four biomarkers of cell death (sFasL, CXCL10, ROC-0.98) [12]. Transcriptome sequencing of SARS-CoV-2 patient’s lymphocytes showed activation of apoptosis and p53 signaling pathway which may account for the observed lymphopenia [136]. A recent study showed up-regulation of IRF1, TP53, and CASP3 apoptotic-related genes in SARS-CoV-2 infected T-cells, suggesting that XAF1, TNF, and Fas apoptotic pathways may be implicated in lymphopenia [137]. André et al. reported an elevated level of both CD95 expressions on T-cells and FasL as well as up-regulation of Bax and Bak transcripts in the plasma of COVID-19 patients. Both the disease severity and CD4^+^ T-cells depletion were directly correlated with lymphopenia and higher levels of CXCL10 biomarker [12]. Another study compared the metabolic profile of immune cells in SARS-CoV-2 patients with other viral infections. A distinctive subpopulation of T-cells was detected and showed an increased expression of the mitochondrial membrane proteins Voltage-Dependent Anion Channel 1 (VDAC1). Activation of this protein mediates T-cells apoptosis through their mitochondria. Targeting the oligomerization of VDAC1 or preventing caspase activation thus protects T-cells [124]. Additionally, SARS-CoV-2 infection was shown to up-regulating T-cells oxidative stress responses, involving PFKFB3, BNIP3, FOS, PDK1, JUN, BHLHE40, GADD45B, and DDIT4. HIF-1a cascade was then activated, acting as an indirect player to elicit T-cell apoptosis [5]. SARS-CoV-2 infection of PBMCs in vitro could cause circulating T-cell apoptosis, however only 6.7% of CD4 ^+^ and 2% of CD8 ^+^ T lymphocytes tested positive for caspase 3/7. Therefore, the authors concluded that apoptosis induction occurs independently of viral replication in these cells and may be induced by proinflammatory cytokines released by inflammatory monocyte cells [138].

#### Hematopoiesis and secondary lymphoid organs dysfunction

**Fig. 3:**
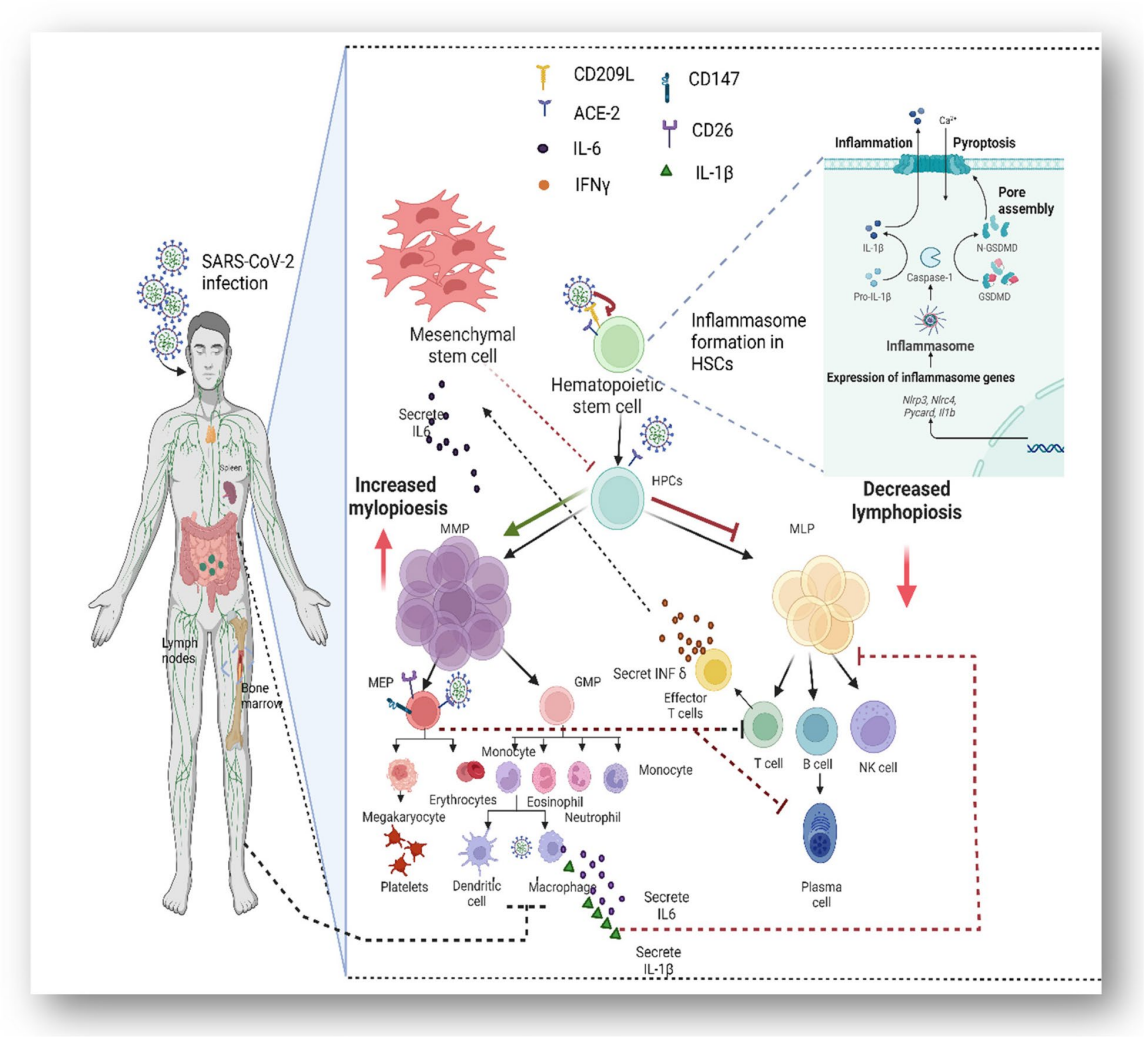
Possible mechanisms of hematopoiesis and secondary lymphoid organ malfunction in SARS-CoV-2 infection. (1) direct viral infection to HSCs/ HSPCs via ACE2 dependent or/and independent manner, (2) inflammation triggered by viral infection results in assembly of NLRP3 inflammasome, which in turn leads to caspase 1-dependent release of the pro-inflammatory cytokines IL-1β and IL-18, and to gasdermin D-mediated (GSDMD) pyroptotic cell death [156], (3) effector cytotoxic T-cells (CTLs) release INFγ upon viral infection that is in turn promotes IL-6 secretion by MSCs, leading to boosting myeloid lineage differentiation at the expense of lymphoid lineage differentiation, (4) in the lymph nodes and spleen, SARS-CoV-2 may directly infect macrophages and dendritic cells, causing tissue damage and indirect lymphocyte depletion due to enhanced pro-inflammatory cytokines IL-6 and IL-1β secretions, (5) erythroid progenitors express several co-receptors, including ACE2, CD147, and CD26, allowing infection by SARS-CoV-2. They also cause immunosuppression of the proliferation of CD8^+^ T, CD4^+^ T, CD3^+^ T and B cells of the adaptive immune system in COVID-19 patients [151].Abbreviations; MLPs, multi-lymphoid progenitors; GMPs, granulocyte-macrophage progenitors; MMPs, multi-myeloid progenitors; MEPs, megakaryocyte erythroid progenitor. Created by Biorender.com

Patients with severe infected SARS-CoV-2 were reported to suffer dysregulated hematopoiesis [17, 140], (Fig. 3). However, the question is whether SARS-CoV-2 directly affects hematopoiesis by infecting hematopoietic stem/progenitor cells (HSPCs) resident in the BM is still debatable. Hematopoiesis is an orchestrated process that controls the expansion and differentiation of HSPCs into different blood forming cell types [141] Zheng et al. reported that 63.4% of isolated human BM cells could be prone to SARS-CoV-2 infection [142]. Although ACE2 gene expression was increased in BM cells, their receptors almost showed negative interaction with the spike receptor binding domain (S-RBD). SARS-CoV-2 may thus infect BM cells in an ACE2-independent manner via another receptor. CD209L was found to interact with S-RBD and this complex could heterodimerize with ACE2 receptors, suggesting that CD209 may be a potential entry route for viral infection [143]. Over-expression of ACE2 was reported in HPSCs and hematopoietic stem cells (HSCs) derived from human umbilical cord blood (UCB), while some other mature immune cells showed lower expression [144]. Interestingly, when HSCs/human progenitor cells (HPCs) were exposed to SARS-CoV-2 spike protein, the differentiation capacity of the multipotent lymphoid progenitors (MLP) to T-lymphocytes, B-lymphocytes and NK cells was reduced [144]. Similarly, a very small population in the UCB CD133^+^CD34^+^Lin^−^CD45^−^ cells known as very small embryonic-like stem cells (VSELs) was reported to express the ACE2 receptors and interact with its spike protein [145]. In addition to the proposed direct infection of HSPCs by SARS-CoV-2, it can indirectly impact HPSC function, survival, and renewal capacity leading to perturbed hematopoiesis. NLrp3 inflammasomes were observed to be hyperactivated in HSCs upon the viral infection resulting in caspase-1 activation and subsequent cell death by pyroptosis [146, 147]. To examine whether HSCs contribute to abnormal blood profiling in COVID-19 patients, a study carried out a single-cell RNA sequencing on samples collected from bone marrow and peripheral blood of six patients [17]. In this study, HSCs of severe COVID-19 patients were arrested in the G1 cell cycle phase and were predisposed to apoptosis. There was also reported significant reduction of lymphoid progenitors, accumulation of immature myeloid progenitors, and up-regulation of ETS2, FLI1, SPI1, LMO4, and GATA2 transcription factors, all of which are important in determining the HSCs fate [148]. During acute viral infection, specific effector cytotoxic T -cells (CTLs) secrete IFNγ, which in turn induce the release of hematopoietic cytokines such as IL-6 from bone marrow-mesenchymal stromal cells (MSCs). IL-6 production reduced the expression of specific transcription factors Cebpα and Runx-1 from early hematopoietic progenitor cells and eventually increased myeloid lineage differentiation [149]. Consistent findings were reported on skewed differentiation of HSPCs toward myeloid precursors in the BM by interferon-γ (IFNγ) secretion [150]. In another study, a specific population of erythroid cells (CECs) that express CD71^+^ was enriched in the blood of severe or moderate COVID-19 patients [151]. The frequency of these cells was negatively correlated with the count of T and B lymphocytes. In particular, the CD45^+^ subpopulation of CECs was found to co-express ACE2, TMPRSS2, CD147, and CD26, and thus can be directly infected with SARS-CoV-2, but also suppress the adaptive immune responses [151]. In addition to hematopoietic progenitors, SARS-CoV-2 was reported to directly infect macrophages and dendritic cells residing in the lymph nodes and the spleen, resulting in hyperproduction of pro-inflammatory cytokines IL-6, IL-8, and IL-1β, and causing tissue damage [152, 153]. As evidenced by splenic atrophy and lymph node necrosis, the virus may also destroy these lymphoid organs, leading to indirect lymphocyte depletion and lymphopenia [154, 155]. Taken together, accumulating evidence suggests that direct viral destruction and/or indirect perturbed function of the HSCs/ HSPCs cell compartments play a role in COVID-19 pathogenesis and may explain the variability in the patient responses to the viral infection.

#### Lipid rafts and virus entry

Lipid rafts are a set of glycosphingolipids, cholesterol, and protein receptors embedded in lipid microdomains within the cellular plasma membranes [157].These microdomain centers function by assembly of signaling molecules and protein-receptor interaction leading to signal transduction and activation of many vital processes. These include membrane protein trafficking and membrane fluidity, in addition to receptor trafficking [157, 158]. Even though lipid rafts exist in an organized confirmation and are tightly arranged compared to the neighboring bilayer, they are neutral and float freely in the membrane bilayer [159]. Lipid rafts include two types, the first is planer, also termed glycolipid or non-caveolar, which is continuous with the plane of the plasma membrane and has nondistinctive morphology [160]. The second includes flask-shaped caveolae that contain caveolin proteins and is the most common in the lipid rafts structures [161]. Depending on cholesterol, sphingolipids, and proteins-enriched lipid rafts, the plasma membrane is more viscous and rigid than the rest [162]. These inflexible microdomains in the cellular membranes function as scaffolds for numerous receptors, which are proposed to interact with the spike protein on the SARS-CoV-2 virus [163–167]. Many viruses such as HIV, Ebola, and Influenza virus use host lipid rafts as an entry point, as they retain a high concentration of receptors used to bind and guide pathogen internalization through endocytosis machinery. Rafts also serve as a platform to facilitate pathogen assembly and exit from the host cells, and its transfer from one cell to another [168]. A study by Wang et al. reported the dependence of Avian retrovirus infection on the integrity of cholesterol-rich lipid rafts, unraveling their significant role in virus entry, assembly, and release. This was achieved by lowering cholesterol, yielding fragile and non-intact lipids [169, 170]. Understanding of lipid rafts’ chemistry provides insight for developing mechanistically designed therapeutics to modulate the removal of cholesterol from them. This removal or alterations in lipid raft mobility will disrupt their regulated signaling pathways, a change that may be used in eliminating the receptors needed for SARS-CoV-2 infection, such as ACE2 [171–173]. Notably, lipid raft-dependent endocytosis that was reported in the earlier SARS-CoV strains as a novel pathway (both clathrin- and caveolae-independent), may contribute to mediating specialized endocytic pathway [174]. In the endosome, the SARS-CoV S protein is activated via pH-dependent protease cathepsin, unleashing the viral RNA in the cellular cytoplasm and resulting in virus amplification [175, 176]. Internalization of virus was reported to be mediated by either syndecan, one of the proteoglycan transmembrane protein family, or heparan sulfate and ACE2, located on the cell surface [177, 178]. In the case of T-cells, ACE2 is barely expressed; hence the question is whether other receptors are recruited to the lipid rafts or whether virus entrance may utilize lipid raft-dependent endocytosis pathway like SARS-CoV. For instance, CD147 is abundantly expressed on activated T-cells [179], the SARS-CoV-2 interaction with CD147 initiates the endocytic mechanism modulated by clathrin-independent endocytosis [180, 181]. Rab5 is a crucial regulator of endocytosis and is located at the early endosome [182]. The co-localization of CD147, Rab5, and S protein was detected in BHK-21-CD147 transfected cells and lung tissues derived from COVID-19 patients [181]. Besides, caveolar/lipid raft-and cytoskeleton-dependent endocytosis were revealed to contribute to the entry of SARS-CoV-2 pseudovirus via Arf6-mediated CD147 endocytosis [183]. Similar to SARS-CoV, SARS-CoV-2 could be using lipid rafts for its independent entry into the host cells [171]. Receptors on cholesterol-rich lipid rafts also play a critical role in regulating inflammation during SARS-CoV-2 infection [184, 185]. It is also worth mentioning that both toll-like receptors (TLRs) and CD147 play an important role in host cell entry and activation of the innate immune response against SARS-CoV-2 [184, 186]. Cholesterol-rich lipid rafts recruited those receptors as a platform to allow the binding with the viral S protein, and may contribute to regulating immune responses [187, 188]. Targeting cholesterol-rich lipid rafts has thus been proposed to regulate the immune response and develop anti-SARS-CoV-2 therapeutics [173]. Moreover, Staffler et al. showed re-establishment and reconstitution of lipid rafts resulting in T-cell receptor (TCR) stimulation, which was inhibited after the blockage of CD147 receptor. By triggering CD147, GPI-robust CD-48 and CD-59 co-receptors rearrangement are activated in the lipid rafts of T lymphocytes. These alterations in lipid rafts combined with TCR-dependent T-cell proliferation selective inhibition suggest a negative feedback loop to modulate lipid rafts, and ultimately CD147 receptor signaling inhibition [15, 23, 189]. In lieu of requiring receptors, SARS-CoV-2 virions can infect cells through the non-endocytic pathway (fusion). Although more investigations are required, it is possible that adhesion molecules, viral proteins, and direct cell-to-cell contact at infected cellular junctions—similar to HIV—will mediate infection [190, 191]. These proteins are known as virological synapses that are assembled by the polarized cytoskeleton. in addition to HIV, human T-cell lymphoma-leukemia virus type 1 (HTLV-1) infects the cells via cell-cell fusion and syncytia formation. The internalization process could be regulated by the released budding virions in the space between the closely interacting cells or cellular fusion into the T-cells to allow efficient propagation between them [192]. Moreover, this process could protect viruses from immune system actions and could be the implemented route to the high infection rate of SARS-CoV-2 in *vivo* [193]. Similarly, SARS-CoV-2 exhibited in vitro transmission through cell-cell contact, mediated by S protein. Interestingly, treatment of co-cultured cells with endosomal entry inhibitors halts cell-to-cell transmission, implicating endosomal membrane fusion as an underlying mechanism [191]. However, further experimentations are required to determine whether targeting lipid rafts to decrease their viscosity can present a faithful viral infection recapitulation [194] .

### Direct viral infection to lymphocytes via cell surface receptors

Although there is no robust evidence for direct causality, SARS-CoV-2 RNA and/or antigens have been detected in multiple immune cells including T-cells from autopsy studies [195–197]. It was suggested that uptake of the SARS-CoV-2 particles does not necessarily result in productive viral infection inside the lymphocytes; instead it might lead to elevated production of granzyme, perforin, IFN-γ, IL-17, and IL-2, causing T-cells exhaustion and death [197, 198]. Moreover, single-cell transcriptome analyses of COVID-19 patients’ samples revealed a sizable proportion of virus-positive cells lacking ACE2 expression, and as such, the mechanism by which the virus entered these cells is still unclear [197, 199, 200]. This knowledge gap suggests a spike-ACE2-indpendent infection to T lymphocytes through other receptors [5] (Table 1.). A recent computational study identified a range of molecules, including adhesion molecules, chemokine receptors, and leukocyte surface molecules, as potential candidates that can interact with spike-RBD. These molecules are expressed on most of the immune cell lineage. The findings showed that ACE2 had lower binding affinities with spike-RBD than XCR1,CD26, CD2, CD7, CD56, CCR9, CD150, CD4, CD50, and CD106 [201] Although several of these molecules such as CD2, CD7, and CD4 are expressed by T- cells and even operate as markers of T-cell subpopulations, experimental studies are still required to determine the amino acids involved in the interactions with the spike-RBD. Of note, many viruses utilize not only one, but multiple uptake receptors. Predicting the possible entry route would provide a better determination of the viral pathogenicity and point out potential new therapeutic targets.

**Table 1:** Proposed cell receptors for SARS-CoV-2 entry to T-cells

Viral entry/receptor	Receptor type	Ligand(s)	Expression in T lymphocytes	Ref.
**ACE2**	Type-I integral membrane protein	A homodimer of ACE2 interacting with two S protein trimers of SARS-CoV-2 via its receptor binding domain (RBD)	Low expression level in T-cells as to the most of the human peripheral blood-derived immune expect for tissue macrophages, such as alveolar, Kupffer, and microglia.	[202, 203].
**TLRs**	Type-1 transmembrane receptors	TLR7/8 sense SARS-COV-2 nucleic acid	Widely expressed in the innate immune cells, mainly dendritic cells, neutrophils, and monocyte/macrophages and on effector T-cells	[20, 204, 205]
**CD147**	Type-1 transmembrane glycoprotein	CD147C-terminal domain interacts with the spike RBD and with several ligands such as cyclophilins, MCTs, Caveolin-1, integrins and induce cellular MMPs.	Expressed on all leukocytes, platelets, and highly expressed on activated T -cells	[181, 206, 207]
**Neuropilin 1 -NRP1**	Type-I transmembrane protein	Interacts with several ligands, including semaphorins 3a and 4a, vascular endothelial growth factor (VEGF) and transforming growth factor-β (TGF-β),	Although NRP1 is expressed on olfactory and respiratory epithelial cells it also expressed on CD4^+^ regulatory T (Treg) cells, and CD8^+^ T	[22, 208]
**AXL**	Tyrosine-protein kinase receptor	Interacts with the N-terminal domain (NTD) of SARS-CoV-2 S.	Expressed on monocytes, macrophages, and dendritic cells.	[209]
**LFA-1**	Integrin acts as an adhesion molecule.	SARS-CoV2 ORF7a interacts with LFA-1.	Widely expressed on the surface of many leukocytes and activated T- cells	[21]
**CD209L**	A member of the C-type lectin (CTLDs) superfamily.	Interacts with S-RBD and complex heterodimerizes with ACE2 receptors	Expressed in human type II alveolar, endothelial cells of lung, liver, lymph nodes, dendritic cells, tissue-resident macrophages, and blood mononuclear cells	[143, 210].
**CD26**	Surface glycoprotein with known Dipeptidyl peptidase (DPP4)	It interacts with coronavirus (MERS-CoV) not SARS-CoV-2	Expressed in epithelium and immune cells except B cells	[211]
**ASGR1 and KREMEN1**	ASGR1: Asialoglycoprotein receptor 1 KREMEN: kringle containing transmembrane protein 1	ASGR1 and KREMEN1 bind to both the RBD and NTD	Expressed mainly on epithelium and hepatic parenchymal cells and at much lower levels in immune cells a subpopulation of regulatory T lymphocytes (Tregs) express KREMEN1	[212–215]

### I-CD147 receptor, structure and tissue tropism

**Fig. 4:**
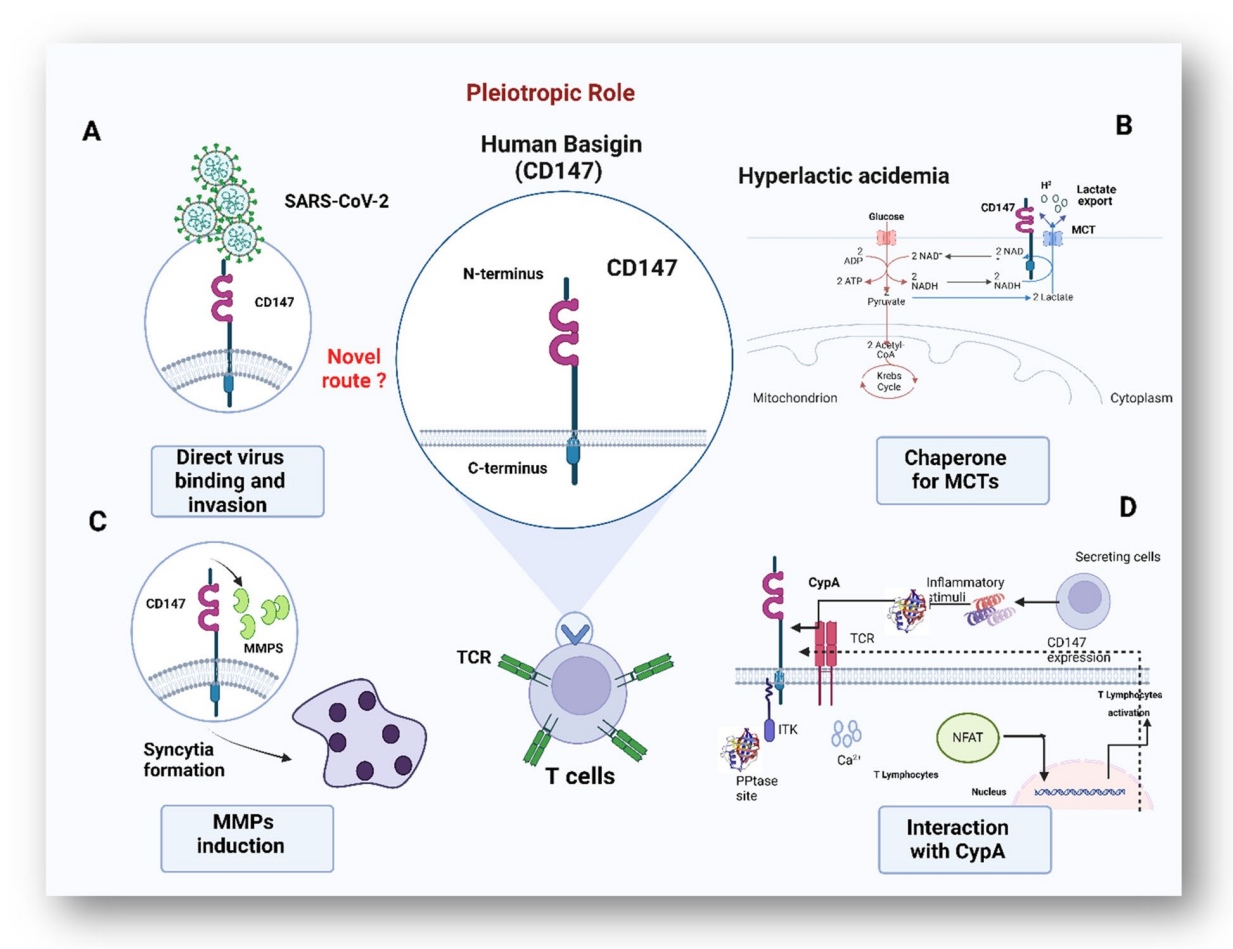
Multiple interactions of CD147 with different partners such as MCTs, MMPs and cyclophilin facilitate the viral infection. **A** The upstream event of direct virus binding and invasion are not well characterized (represented as ‘?’). CD147 directly binds with the viral spike protein to enter to host cells. **B** CD147 acts as the major chaperone protein for MCTs increasing lactate export leading to increased blood lactate levels and disease severity in COVID-19 patients. **C** SARS-CoV-2 binding with CD147 increases synthesis of MMPs, allowing syncytium formation and escape from immune cells. **D** Cyclophilin binds to and activates CD147, leading to better interaction between the viral S protein and CD147. CypA was secreted in response to inflammatory stimuli to interact back with CD147 on the surface of T-cells. CypA makes a stable complex with Tyrosine-protein kinase (ITK) in the cytoplasm of T-cells and activates nuclear factor of activated T cells (NFAT) by downstream signaling cascade. NFAT eventually regulates gene expression and activates T-cells and cytokine generation

CD147 or EMMPRIN, is a type-1 transmembrane glycoprotein that belongs to immunoglobin superfamily (IgSF), characterized by expressing variable domains in their sequence patterns [216]. Primarily, CD147 was known as Basigin when first discovered by Biswas [217], and characterized as a tumor surface antigen, stimulating collagenase production in fibroblasts in the tumor niche [218]. Later, CD147 was found to elicit a major role in the expression of MMPs [219, 220], therefore, the term extracellular matrix metalloproteinase inducer (EMMPRIN) was eventually used. Two isoforms of CD147 were identified; the first is known as the primary or classical form (CD147 Ig1-Ig2), and the second expresses an additional extracellular domain CD147 Ig0-Ig1-Ig2, specifically expressed on retinal cells [29]. In 2011, Redzic and colleagues resolved the molecular structure of Ig0 domain of the retinal specific CD147 type using X-ray crystallography [216]. CD147 was first found to play a role in extraembryonic tissues and fetal development [221]. It has also roles in spermatogenesis [222, 223], tissue remodeling, wound healing [224] and immune-mediated responses and cytokine release. The structure of human CD147 consists of 269 amino acids distributed into three domains: an extracellular domain, a transmembrane domain, and a cytoplasmic domain. Molecular studies showed the presence of a conserved glutamic acid residue (Glu218) in the middle of CD147 hydrophobic transmembrane domain. The existence of a charged amino acid in this region is an uncommon structural feature for proteins which span the membrane only once (such as CD147) due to the high energy that accompanies their presence in the middle of the lipid bilayer of the membrane. Therefore, it is believed that this glutamate residue plays a critical role in the interaction of CD147 with other proteins within the plasma membrane [225]. CD147 works with many binding partners on cell surface to perform various biological functions [226]. This functional versatility of CD147 is due to its ability to interact with multiple partners, especially extracellular CyPs, MCTs 1–4, caveolin-1, integrins α3β1 and α6β1 (Fig. 4). The expression of CD147 is noticeable in the brain, gastrointestinal tract, genital organs mainly the testis, kidneys, and muscle tissues specifically heart muscle. It was also highly expressed in intestinal epithelial cells, although its function within these cells remains unclear. Enhanced expression and specificity of CD147 was reported in syncytiotrophoblasts and cytotrophoblasts [13]. CD147 was detected in several immune cells including granulocytes, monocytes, dendritic cells, T-cells and natural killer cells [13], which suggest the involvement of the protein in inflammatory responses and immunity [227]. Significant expression of CD147 has been also reported in many inflammatory diseases, such as lung inflammatory disease [228], rheumatoid arthritis [229], systemic lupus erythematosus(SLE) [230], ischemic stroke [231] and atherosclerosis [232]. Therefore, the local pathology, affected cells, the microenvironment, and the expression of co-molecules all affect the outcome of CD147 activation and function.

### II- CD147: a modulator of the immune response and inflammation

CD147 was initially identified as a T-cell activation-associated antigen and given the name M6 Ag [23, 189]. In addition to acting on mature T-cells, CD147 also controls the development and maturation of early thymocytes [233]. CD147 is modestly expressed on dormant T-cells, while is highly up-regulated in activated cells [234–236]. Cyclophilins exist inside the cells, but are also released upon inflammatory stimuli and they initiate chemotactic activity for some inflammatory cells such as neutrophils, eosinophils and T-cells [234, 237]. These activated T-cells migrate more readily to CypA than resting cells, indicating cyclophilin-CD147 interactions [234]. CD147 regulates T-cell activation by forming a complex with the plasma membrane calcium ATPase isoform 4 (PMCA4) to dampen IL-2 production via NFAT and NF-κB in leukemic Jurkat T-cells and primary CD4 ^+^ T-cells [238]. CD147-CyPA was also found to contribute to immune modulation during viral infection. The signal axis of S protein-CD147-CyPA causes cytokine storm generation in severe cases. Through CD147, SARS-CoV-2 infection triggers the JAK-STAT pathway, which induces the production of cyclophilin A (CyPA), which in turn interact back with CD147 and activates MAPK pathway, leading to positive feedback production of cytokine [25]. Targeting CD147/cyclophilin-CD147 interaction may thus provide a novel approach for alleviating severe inflammation associated with SARS-CoV-2 infection [239]. The confirmation of CD147 role in inflammatory disease was highlighted by examining the efficacy of some Cyclosporine A benzimidazole derivatives in blocking extracellular cyclophilins in CD147-deficient mice. It was reported that CD147 is solely responsible for the inflammatory disease which involves leucocyte chemotaxis [240]. In addition, it was found that the use of anti-CD147 mAb to block the interaction of cyclophilins with CD147 markedly decreased the levels of inflammatory cells at the site of inflammation [241]. Interestingly, CD147 is upregulated in patients with obesity and diabetes. Obesity-related chronic and systemic inflammation can lead to insulin resistance (IR), β-cell dysfunction, and type 2 diabetes mellitus (T2DM) [242]. When compared to controls, obese diabetic subjects had significantly higher levels of CD147 in their blood and the glycosylated CD147 protein in their visceral adipose tissue (VAT) [243], which may help to explain, at least in part, why these comorbidities increase complications and mortality in COVID-19. An anti-inflammatory macrophage phenotype (M2) was shifted in the CD147 knockout mice, which is responsible for the development of atherosclerotic plaque [244]. CD147 thus presents a novel potential target for the treatment of many inflammatory diseases.

### III- CD147 and infectious diseases

The role of CD147 in mediating viral entry has been shown in many infectious diseases. For instance, CD147 was reported to facilitate HIV-1 entry into T-cells [245], and antibodies to CD147 inhibited HIV-1 entry and led to the eventual inhibition of viral reverse transcription. The mechanism was attributed to interaction of this receptor with virus associated cyclophilin A (CyPA) and regulation of an early step in HIV replication. CD147 was also reported to act as a functional entry receptor for measles virus on epithelial cells via virion associated CypB, and to mediate the entry of cytomegalovirus (CMV) into endothelial and epithelial cells [246]. It was also reported that measles virus takes advantage of CD147 for viral entry in host cells with the aid of cyclophilin B (CyPB) [247]. *Plasmodium falciparum* takes advantage of the erythrocyte receptor CD147 by binding of the parasite ligand PfRh5. This interaction plays a role in the blood growth stage during infection [248, 249].*Neisseria meningitidis i*nteracts with CD147 host receptor through the meningococcal pilus components PilE and PilV. Blocking this interaction prevented blood vessel colonization in human brains [250, 251]. CD147 was found to be associated with viral pathogenic infections such as hepatitis B (HBV) and C viruses (HCV) and  Kaposi's sarcoma-associated herpesvirus9(KSHV) [29]. Upon infection with human Cytomegalovirus (HCMV), upregulation in the secretion of cyclophilin A ligand suggested its direct interaction with CD147 [252]. The antiviral immune response elicited against HCMV was believed to occur through the activation NF-κB and IFN-β signaling pathways that was significantly decreased after CD147 knockdown [253]. In another study, CD147 was found to indirectly promote the viral entry of HCMV into epithelial and endothelial cells through cellular proteins [246]. HCMV targets CD147 using miR-US25-1-5p in order to escape from the innate immune response [252, 253]. Similarly, CD147 and NOD2 interaction was reported to have a role in bacterial invasion of epithelial cells as CD147 enhanced the invasion of *Listeria monocytogenes* of host cells [254]. CD147 was found to play a functional role in facilitating SARS-CoV invasion of HEK 293 cells, and CD147 antagonist peptide-9 inhibited SARS-CoV infection [255].The similarity between SARS-CoV and SARS-CoV-2, the causative agent of COVID-19 urged investigating the role of this receptor in SARS-CoV-2 entry into the human cells.

### IV-CD147: SARS-CoV-2 and new emerging variants

As evidence for direct invasion of SARS-CoV-2 via CD147, virions were detected in the lymphocytes infiltrating the lung of infected patient [181, 256]. Interestingly, CD147 expression was detected in both CD4^+^ and CD8^+^ T-cells of SARS-CoV-2 infected patients blood [181]. In SLE patients, elevated expression of CD147 on CD3^+^ T-cells was also found to increase vulnerability to SARS-CoV-2 infection and associated lymphopenia [257]. To understand the mechanism of SARS-CoV-2 entry via CD147, a recent *in-silico* analysis predicted the interaction of the virus’ spike external subdomain, specifically the groove between the short antiparallel β strands 1’ and 2’, and the small α1 helix, with the C-terminal domain of the CD147 receptor. Ligand/receptor complex was subjected to molecular dynamic simulation (MDS) and binding free energy calculation. The residues of CD147 at the interface achieved a significant stability over time and reached 1 Å after 60 ns, indicating significant interaction [206]. A study by Wang et al. discovered CD147 as new route for SARS-CoV-2 invasion in ACE2-deficient T-cells (BHK-21 cells). Constant binding and localization of spike protein-RBD and CD147 receptor was achieved using co-immunoprecipitation and immuno-electron microscopy and validated by Meplazumab antibody blockade of the receptor [181]. Since ACE2 binding promotes proteolytic activity as serine proteases (TMPRSS2) induce S protein priming, activation and enabling virus–host membranes fusion [258, 259], the question arises to whether CD147 utilizes TMPRSS2 for virus priming and entry, similar to ACE2. Interestingly, neither ACE2 nor TMPRSS2 were expressed or co-localized on the surface of activated or inactivated human or Jurkat T-cell, and SARS-CoV-2 infection was suggested to be independent of both molecules [5, 256]. Additionally, another non-fusion pathway through clathirn-independent pathway was confirmed to promote virus entry without the necessity for TMPRSS2, as both S-RBD and CD147 molecules were co-localized in the cellular endosome [207]. It is important to note that metalloproteinase activation of SARS-CoV-2 S represents a third entry pathway in cells expressing high MMP levels [260, 261]. In particular, MMP2/9 can activate SARS-CoV-2 S fusion activity, but not that of SARS-CoV-1. This route of entry required cleavage at the S1/S2 junction in viral producer cells and syncytia formation. Interestingly, metalloproteinase inhibitors reduced replicative Alpha variant, while Omicron exhibited reduced metalloproteinase-dependent fusion and infection [260]. As CD147 regulates and induces MMPs expression during certain pathological condition [220, 261], SARS-CoV-2 binding with CD147 would also increase synthesis of MMPs, allowing not only viral entry but also efficient propagation within syncytia and escape from the immune cells [191]. It is thus reasonable to deduce that CD147 and MMPs might promote syncytia formation during SARS-CoV-2 infection, although this postulation has not been fully resolved and requires further investigations. Despite the above evidence, other *in-vitro* studies showed no visible interaction between CD147 receptors expressed on human cells and the recombinant forms of the SARS-CoV-2 spike protein [262, 263]. In particular, Shilts and colleagues further reported that knocking down CD147 in Calu-3 cell line, which also expresses high levels of ACE2 receptors, had no effect on the cell sensitivity to SARS-CoV-2 infection [263]. In contrast, Xu et al. [24] created a pseudotyped SARS-CoV-2 virus model system that may infect cells that do not overexpress ACE2 receptors, potentially allowing the discovery of novel receptors for viral entry. Their findings imply that the infection of epithelial and other immune cells that exhibit low level of ACE2 may be caused by the interaction of a distinct area of the spike protein with CD147 [24]. Later, based on earlier findings [264], Fenizia et al. proposed that the recognition of SARS-CoV by CD147 receptor is dependent on CyPA binding and that a similar mechanism holds true for SARS-CoV-2 [255]. For validation, CD147 receptors were knocked out in the pulmonary cells (Calu-3 cell line). Although binding of CD147 to CyPA played no role in SARS-CoV-2 infection, the ACE2 expression levels were reduced and the viral infectivity of these cells was also compromised [264]. Further studies confirmed that SARS-CoV-2 infection of T lymphocytes was independent of ACE2 expression, suggesting another receptor for the viral entry through lymphocyte function-associated antigen-1 (LFA-1) [6]. CD4 molecule also facilitate SARS-CoV-2 entrance into CD4^+^ T-cells, but not CD8^+^ T-cells, as it was detected in the blood of severe COVID-19 cases [265]. It is worth noting that neither SARS-CoV-2 or its variant delta caused fibroblast activation and eventually fibrosis in humanized CD147 transgenic mouse model [266]. Deletion of CD147 in fibroblasts inhibited fibroblast activation and reduced susceptibility to bleomycin-induced lung fibrosis. Meplazumab, a CD147 antibody, was able to slow the development of lung fibrosis caused by SARS-CoV-2 by preventing the buildup of activated fibroblasts and the generation of extracellular matrix (ECM) proteins [266]. These discrepancies in research findings due to the experimental design and methodologies support the need for additional work and more data collection from clinical studies to explore the role of CD147 in SARS-CoV-2 infection and pathogenicity. However, for new emerging SARS-CoV-2 variants, it is yet unclear whether S protein harboring new mutations could utilize CD147 in viral infectivity and spread. Coronavirus are known to exhibit substantial degree of antigenic drift, often resulting in adaptive immune escape [267]. When Geng and coworkers blocked CD147, viral entry and cytokine storm triggered by SARS-CoV-2 or its new variants—alpha, beta, gamma, and delta were significantly reduced [25]. The emerging Omicron is the most mutated SARS-CoV-2 variant in term of higher reinfection rate, immune evasion, resistance to the neutralizing activity of vaccines and convalescent serum [268, 269]. Recently, it was discovered that the S protein of the Omicron variants may generate more CD8^+^ T-cell epitopes than the Delta variant. These epitopes have the potential to elicit strong CD8^+^ T-cell responses. In humanized transgenic mouse models, CD147 mediated Omicron infection induced exudative alveolar pneumonia [270]. Identification of new emerging variant and their targeted route of infection may help our understanding of SARS-CoV-2 cell tropism, pathogenesis and vaccination.

### V. CD147 as a drug target

As discussed earlier, several studies have demonstrated the role of CD147 in the entry of SARS-CoV-2 to the host cells. And despite the need for further validation and investigation to understand the molecular mechanisms of the viral interaction with this immunoglobulin, researchers have already initiated the quest for ligands capable of disrupting this interaction. Recently, humanized anti-CD147 antibody, Meplazumab, was examined in a large-scale phase II clinical trial to assess the safety and efficacy of viral-neutralizing mAbs for treating COVID-19 patients. Unfortunately, the authors could not reach a conclusion regarding this modality and suggested long term future studies to evaluate the clinical usefulness of these mAbs [271, 272]. Interestingly, many FDA-approved drugs were recently repurposed for inhibiting SARS-COV-2 cellular entry via disrupting its interaction with CD147. These include some beta-blockers, Ivermectin, and statins [273]. It was reported that beta-adrenergic blockers down-regulate CD147, and thus, could reduce the availability of this receptor for viral entry, albeit not yet validated by the appropriate clinical trials [274]. Statins are also involved in the downregulation of CD147 via blocking N-glycosylation and isoprenylation of the protein. In a rodent model of atherosclerosis, statins were found to reduce the levels of CD147 and attenuate the plaque susceptibility [275]. Furthermore, most protease inhibitor (prinomastat and marimastat) specific for MMP2 and MMP9, have been shown selective action against SARS-CoV-2 but not against SARS-CoV [276]. Upon clinical validation, these CD147-targeting drugs could be repurposed, at least, as adjuvant therapy in COVID-19 treatment protocols.

### Concluding remarks

Since 2002, humans suffered several coronavirus outbreaks. SARS-CoV was in 2002, MERS-CoV in 2012, finally SARS-CoV-2 since 2019, and still evolving. Hospitalized adults with severely SARS-CoV-2 infection regularly suffered lymphopenia, particularly of the CD8^+^T-cells. To prevent infection, a healthy T-cells response is essential, as T-cells react to at least 30 viral protein epitopes and have prolonged memory, in contrast to antibodies which are less persistent and can only neutralize RBD of the S protein. Furthermore, despite the absence of a specific memory B-cell response in these individuals, SARS-CoV-2-specific memory, CD4^+^T-cells were discovered to endure in the peripheral blood for years after infection. This seems to indicate that the SARS-CoV-specific T-cells response may last longer, showing the importance of the cell-mediated immune response in preventing re-infection. Although the exact cause for lymphopenia is still unclear, several indirect and direct mechanisms can account for this pathology, either alone, or synergistically. Thus therapeutics has been tailored to target specific underlying cause of T-cells inadequacy. For instance, immunosuppressive drugs are seen as a possibly useful option for controlling cytokine storm in patients with poor prognosis. A Phase III clinical trial showed that anakinra (IL-1 blockade) significantly improved the survival without exerting notable side effects among the patients with hyperinflammation and sepsis (NCT number NCT04443881). Asunercept (Phase II clinical trial) is a human fusion protein made up of the CD95 receptor and an IgG1 antibody, typically used for the treatment of solid tumors and hematological malignancies. Asunercept is currently being developed to treat viral infections such as SARS-CoV-2 by speeding up recovery, as lymphopenia and the severity of the COVID-19 disease are correlated (NCT04535674). Baricitinib (IL‐6 receptor blockade) is selective Janus kinase pathway inhibitor with known anti-inflammatory properties. Treatment with baricitinib in addition to standard care (including dexamethasone) had a similar safety profile to standard care and was associated with lower mortality in ICU patients, even though there was no discernible decrease in the overall frequency of disease progression [277]. Despite the concerted efforts of the medical community, the virus will also almost certainly develop additional mutations that may be associated with increased virulence. The novel receptor, CD147 was reported to mediate the entry of emerging new variants— Omicron, alpha, beta, gamma, and delta. Blocking of this receptor by such FDA approved drugs as Azithromycin, Ivermectin, statins, and beta blockers would enhance T-cell immunity against a future re-emergence of SARS-CoV-2. Despite the discovery of various lines of therapies, many questions need to be clarified to accurately target the cause of SARS-CoV-2-associated-lymphopenia. The affinity of the new SARS-CoV-2 variants to CD147 and/or other receptors expressed on T-cells is to what extent? How the next virus variants could escape from the immune surveillance. How to develop therapeutics able to maintain the balance between inhibition of the inflammation and enhancing lymphocyte functions in advanced patients. Immune suppressive drugs as corticosteroids that reduce T-cell response, particularly CD4^+^cells, even at low-dose, may confer increased risk of infection especially in patients with chronic immune-mediated inflammatory diseases (IMIDs). The next pandemic when it strikes—and it probably will—what will be the optimal treatment regime. While the exact cause of lymphopenia remains a puzzle, experts continue to investigate and dig deeper in quest of a potential therapeutics to boost the natural durability of our immune system.

## Data Availability

No datasets were generated or analysed during the current study.

## Abbreviations

TLRs: Toll-like receptors
CyPs: Cyclophilins
MPPs: Matrix metalloproteinases
MTCs: Monocarboxylate transporters
SARS-CoV-2: Severe acute respiratory syndrome coronavirus-2
NK: Natural killer
ACE2: Angiotensin-converting enzyme 2
IFN: Interferon
TNF: Tumor necrosis factor
HIV: Human immunodeficiency viruses
BM: Bone marrow
GMPs: Granulocyte-monocyte progenitor cells
Nsps: Non-structural proteins
VOCs: Variants of concern
TMPRSS2: Transmembrane protease serine 2
NAAT: Nucleic acid amplification testing
ARDS: Acute respiratory distress syndrome
ICU: Intensive care unit
PLA2G1B: Phospholipase A2 group IB
PD-1: Programmed cell death protein 1
Tim-3: T cell immunoglobulin and mucin domain 3
VDAC1: Voltage-Dependent Anion Channel 1
CSS: Cytokine storm syndrome
IL-6: Interleukin 6
ROS: Reactive oxygen species
JAK/STAT: Janus kinase/signal transducer and activator of transcription
HIF-1: Hypoxia-inducible factor-1
APAF1: Apoptotic peptidase activating factor 1
OXPHOS: Oxidative phosphorylation
HSCs: Hematopoietic stem cells
MSCs: Mesenchymal stem cells
UCB: Umbilical cord blood
HPCs: Human progenitor cells
HSPCs: Hematopoietic stem/progenitor cells
ASGR1: Asialoglycoprotein receptor 1
KREMEN: Kringle containing transmembrane protein 1
SLE: Systemic lupus erythematosus
HIB: Hemophilus influenzae B
T2DM: Type 2 diabetes mellitus
VAT: Visceral adipose tissue
VEGF: Endothelia growth factor
Cyp: Cyclophilin
HCMV: Human Cytomegalovirus
PBMC: Peripheral blood mononuclear cells
MDS: Dynamic simulation
ECM: Extracellular matrix
